# Understanding and Cultivating Effective Listening: A Dialectical Theory of the Tensions Between Intuition and Behavior

**DOI:** 10.3390/bs16040572

**Published:** 2026-04-10

**Authors:** F. K. Tia Moin, Guy Itzchakov, Netta Weinstein

**Affiliations:** 1 School of Psychology and Clinical Language Sciences, University of Reading, Reading RG6 6UR, UK; tiamoincoach@gmail.com (F.K.T.M.); n.weinstein@reading.ac.uk (N.W.); 2 Department of Human Services, University of Haifa, Haifa 3498838, Israel

**Keywords:** listening, listening training, active listening, dual-processing

## Abstract

High-quality listening is a multifaceted social behavior, and theories and research concerning listening and how to train people to listen are mixed in terms of listening definitions and recommendations. The current study canvassed lay practitioners’ understanding of optimal listening qualities and training, drawing on a wide range of listening training materials (N = 207) sourced from the World Wide Web. Thematic analysis results were critically examined to systematically position praxis against our current understanding of listening theories. Findings are presented as a “dialectical listening theory,” which posits that at its core, listeners’ behaviors often exist in direct tension with their mindset or intuition. Furthermore, we posit that this tension is amplified when individuals are faced with conversations that conflict with their perspectives or values, making learning to listen challenging in practice. We conclude that high-quality listening requires direct recognition and strategic management of these tensions throughout the listening process and make recommendations based on listening and cognitive theories to inform best practice in listening training.

## 1. Introduction

People know when they are listened to well. Speakers form holistic evaluations of their conversation partners and report with some confidence when they feel ‘listened to’ or not, evaluations that impact their reactions to the conversation (Lipetz et al., 2020). In their conceptual and empirical work, researchers have attempted to address what listening looks like, largely in terms of the relational observed and unobserved behaviors used by the listener (e.g., Kluger & Itzchakov, 2022). Yet researching the nature and outcomes of listening has not been straightforward because approaches to conceptualizing the construct are complex, varied, and fragmented, and have spanned a range of disciplines, including psychology, communication, management, and linguistics (Bodie et al., 2008; Glenn, 1989).

Outside of the research domain, listening is understood to be a fundamental tool that benefits individuals in professions that rely on communication. Those who listen well tend to perform better at their jobs, including sales, healthcare workers, customer service professionals, journalists, and leaders (Drollinger et al., 2006; Harro-Loit & Ugur, 2019; Itzchakov, 2020; Kluger & Itzchakov, 2022; Wouda & van de Wiel, 2014).

Beyond helping people perform better, many professional roles, such as coaching, mentoring, consulting, counseling, and psychotherapy, may rely on listening as a core skill or activity (Burt, 2019; Lai & McDowall, 2014; Rogers, 1942; Stein, 2021). Recognizing the significance of listening as a performance enabler, practitioners have given ample attention to developing listening training to help others improve their listening (e.g., Itzchakov, 2020; Itzchakov et al., 2022b). The sum of knowledge by the lay practitioner is largely untapped in academic research. Still, this knowledge can help researchers develop clearer working definitions of listening and advance listening theory, including addressing gaps in our understanding about how to train people to listen well.

### 1.1. Academic Conceptualizations and Implications of Listening

Several branches of listening research have developed in recent years. For example, listening has been used to improve impaired attention (e.g., in children with autism spectrum disorder; Irwin & Brancazio, 2014), language acquisition (Feyten, 1991), learning and well-being in educational contexts (Rave et al., 2022), and to facilitate relationships with others (Bodie, 2012; Kluger et al., 2021). The current paper is particularly concerned with the latter—interpersonal listening—which is sometimes referred to as “active listening” (Rogers & Farson, 1957) or “active-empathetic listening” (Drollinger et al., 2006) in the academic literature. Interpersonal listening can be understood as “a complex behavior that helps signal involvement or the degree to which participants are enmeshed in the topic, interpersonal relationship, and situation” (Coker & Burgoon, 1987, p. 463).

Several components of interpersonal listening have been identified from listening research, which comprise our understanding of listening to date (Itzchakov & Kluger, 2017; Kluger & Itzchakov, 2022). These include attention (e.g., gazing, focusing, remembering), comprehension (e.g., processing, interpreting, learning), and positive intention (e.g., validating, empathizing, being non-judgmental). These three constructs have been found to have strong positive causal relationships with feeling listened to (Kluger & Itzchakov, 2022). Further downstream, as individuals perceive themselves to be listened to well, they report many greater benefits such as well-being (Itzchakov, 2020; Itzchakov et al., 2022c; Itzchakov & Weinstein, 2021; Trahan & Rockwell, 1999), shared understanding and openness (Itzchakov et al., 2022a), and willingness for future self-disclosures (Weinstein et al., 2021, 2022); for reviews see (Bodie, 2011b; Kluger et al., 2021).

### 1.2. Training People to Listen Well

Researchers have been implementing listening training for the past several decades in work that has attempted to test the outcomes of listening in everyday contexts. The earliest listening training study of which we are aware focused on telephone counselors dating back to the 1960s (Ross & Shoemaker, 1969), and more recently, meta-analytic data from 32 studies show an average moderate effect size on listening behaviors from listening training at r = 0.38, 95% CI [0.30, 0.46], τ = 0.21 (Kluger, 2020). There is some evidence that even relatively brief training can be successful. Training as short as two hours or two days can improve listening behaviors (e.g., Aakre et al., 2016; Davidson & Versluys, 1999; Graybill, 1986; Lisper & Rautalinko, 1996). In a few cases, researchers reported the transfer of training to practical contexts, for example, after listening training was delivered to counseling students (Levitt, 2001) and insurance customer service employees (Rautalinko & Lisper, 2004), their professional practice was enhanced. However, listening training is not consistently effective in creating the intended downstream benefits. For example, Rautalinko and Lisper (2004) reported that customers did not experience different listening as a function of employees receiving training. In a parental communication program, although parents were objectively assessed as showing improved listening and felt more confident and competent, the children did not notice any differences, nor were there downstream effects on children’s well-being (Graybill, 1986). In addition to downstream effects on the intended recipient of listening, another component of listening training that is inconsistently reported is the amount of time taken to train people to listen well. Establishing effective training methods that address time limitations is imperative in practice, and we are still unclear on the optimal amount of time needed to train people to listen well. Recent findings suggest that very brief 10 min training interventions embedded in broader courses may lead to negligible changes (Martin & Butera, 2022), and possibly extended programs yield better results (Rautalinko et al., 2007). It is as yet unclear whether it is difficult to train individuals to listen well or whether the basic approach to training listening needs to be developed.

### 1.3. Present Study Aims

Researchers have developed working definitions of listening, but there is little consensus in the academic literature about what listening entails or how best to develop others’ listening (Weinstein et al., 2022).

Laypeople’s perception can help to expand on broad and multi-faceted constructs (such as listening) to contribute to the scientific discourse, and they can offer academics resolution when concepts under study are intuitive and present within public conversations (Haddock et al., 2022; Schlehofer et al., 2008). Given the wide recognition of listening as an important human ability (Bodie, 2012), listening training resources are publicly shared on the World Wide Web (www), but this content has lived in relative isolation from research and the published literature.

A critical analysis of this information from a lay community of practitioners can provide important information on what tensions or challenges exist in how we define, learn, and apply the listening process in everyday practice, how practitioners address tensions or challenges in vivo, which can then, through a process of theory reconstruction, help to advance our understanding of both theory and practice.

It can be helpful to consider such a communication practice challenge by first defining the core issue or problem, identifying the communication techniques applied in practice to overcome the challenge, followed by a consideration of ideals or philosophies that guide actions to address the challenge (Craig & Tracy, 2014). The findings from these steps of critical inquiry form a normative listening theory that we expect will guide future listening research that is of practical relevance on the one hand; and on the other, informs listening training strategies that broad agreement suggests matter most.

The current research employed thematic analysis (Robinson, 2022) to draw insights into how we understand and develop listening from the large body of practitioner training materials on interpersonal listening. The paper then critically examines the practitioner’s understanding of listening and listening training practices in light of researchers’ current perspectives, using a problem-focused approach inspired by the core principles of Grounded Practical Theory (Craig & Tracy, 2014) as outlined above. Themes were critically examined by identifying practical challenges, problems, or tensions in the learning process, which are noticed and addressed by the listening practitioner, in order to better meet practitioner ideals. Using this approach, we attempted to construct a new, normative theory (rationally define universal principles and values) of listening and learning to inform listening theory, future research directions, and best practice in listening training.

This contribution is especially relevant for workplace communication, where listening shapes how employees, managers, leaders, and colleagues navigate feedback (Itzchakov & Kluger, 2018), disagreement, support, and day-to-day coordination (Vinokur et al., 2024). By clarifying the tensions involved in listening, the present study also offers a framework for designing workplace communication training that goes beyond teaching mere behaviors.

## 2. Materials and Methods

### 2.1. Sourcing Data

A search was carried out on publicly available web content using the search engine Google (accessed via Chrome Browser: Version 110.0.5481.177/178) because of its top ranking and global coverage of over 90% of the web (RapidAPI, n.d.). Search terms included “listening and training,” “listen and training,” “active listening exercises,” “listening courses,” and “listening skills.” These terms were chosen to capture variations in search terms that cover “listening skills training courses.” The term “active listening” was chosen to acknowledge that the term has been broadly applied to listening training since its popularization by Carl Rogers (Kluger & Itzchakov, 2022; Tyler, 2011).

To ensure adequate breadth and depth of qualitative data in the sample size, analysis continued until a point of saturation was reached with themes (Fusch & Ness, 2015). Initially, we analyzed 106 texts, though the analysis revealed that many training materials were outlines only, rather than actual listening training content. Therefore, we doubled the sources to obtain a more sufficient set of data, and the top 207 texts (approximately 15 pages) were sourced from search results and included a broader range of blog posts, articles, training course outlines, and freely available training materials, providing much richer data. Articles and content specific to listening for the purpose of learning a language or educational learning were excluded, as this form of listening is markedly different from the type of listening we are interested in exploring further in this study, namely, listening for human connection.

Search settings were set to the default region (United Kingdom) and English language by Google. Results included organizations and institutes in the United States, United Kingdom, the Netherlands, and Australia, for example, the Center for Disease Control and Prevention (Atlanta, Georgia, United States), AstraZeneca (Cambridge, United Kingdom), PositivePsychology.com (Maastricht, The Netherlands), and Professional Development Training (Sydney, Australia), but there was a limited representation of entities in non-English-speaking countries.

### 2.2. Epistemology and Approach to Thematic Analysis

From a position of critical realism (Bhaskar, 2008), assuming that empirical observations of reality will be subject to some interpretation by researchers, our epistemological position assumed a “hybrid” approach to thematic analysis that incorporated both deductive and inductive measures; known as Structured Tabular Thematic Analysis (ST-TA; Robinson, 2022)—steps of which are outlined in detail in the Supplementary Information (pp. 2–8). The ST-TA method was chosen as it is appropriate for analyzing large quantities of short-text (rather than lengthy interview transcripts) and because the epistemological position of ST-TA is well aligned to this research project (Robinson, 2022), situating itself between the essentialist approach of Boyatzis (1998) and the constructionist approach of Braun and Clarke (2006).

The eight-step ST-TA approach is outlined in detail in the Supplementary Information (pp. 2–8). It describes how the lead researcher and team of coders worked together to rigorously identify the essential, common listening factors in publicly available listening training texts. Analysis began with an a priori set of codes developed from an initial set of sources selected by the lead researcher, who looked for frequently used terms, different terms that referred to the same underlying meaning, and terms that were emphasized within the text (Owen, 1984). A team of coders was then guided through this process for coding and the preliminary codebook and proceeded to add codes inductively in the same way as the lead researcher on a small sample of texts. At this point (and at several other intervals throughout the process—see Supplementary Information for step-by-step procedure), coders met to discuss and calibrate their understanding of codes with the lead researcher’s guidance. A portion of texts (25%) were double-coded (i.e., a second coder analyzed the same text), and discrepancies were identified and discussed between coders. Reflexivity was a part of these discussions, and the coding team acknowledged their collective experience and subjective influence as psychologists with experience in organizational, coaching, and clinical psychology in identifying both codes and themes—any notations and decisions are summarized in the Supplementary Information (pp. 4–6). Qualitative data were interpreted at a semantic level (surface meaning of language). Following critical, reflexive discussion, coders aimed for consensus on the final set of codes and themes (see Supplementary Information for complete tables), before inter-rater reliability (IRR) analyses were calculated for each of the codes (Boyatzis, 1998—see Supplementary Information Table S2 for calculations, pp. 19–22). This guided the final set of themes, sub-themes, and descriptions documented.

## 3. Results

**Figure 1:**
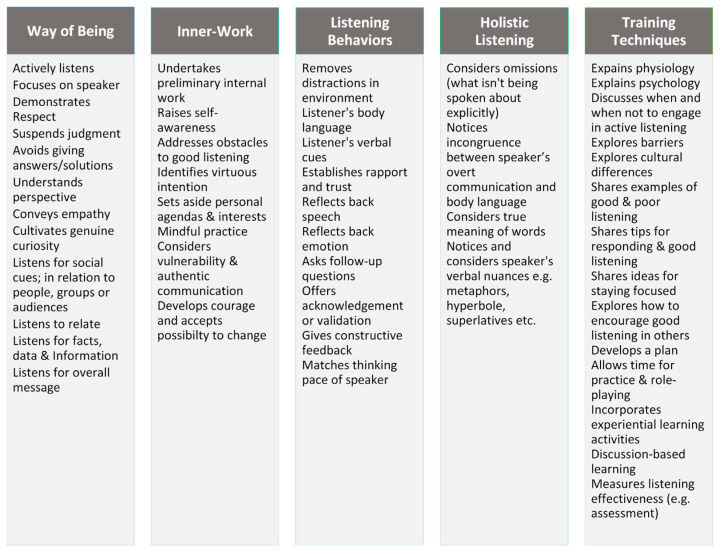
Themes and sub-themes identified from thematic analysis of the data.

**Figure 2:**
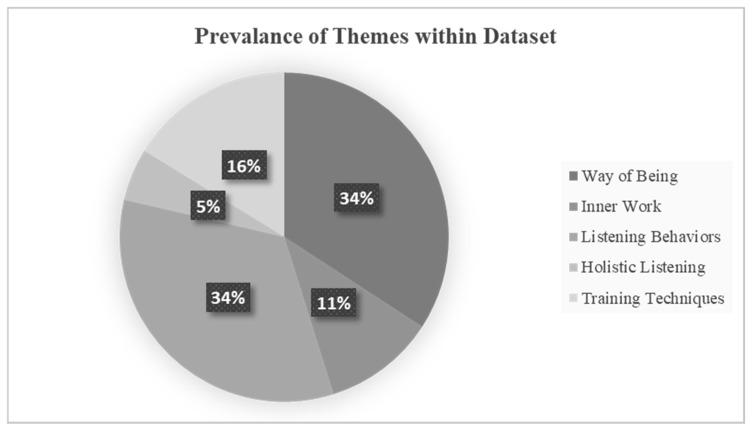
Portion of codes attributed to each theme from the dataset of 207 training texts.

A total of five themes were identified, outlined in Figure 1 and Figure 2 and described further below (refer to Supplementary Information Table S1, pp. 9–18). Way of being and listening behaviors were similarly prevalent at 34% of the analyzed data. Training techniques (16%), inner-work (11%), and holistic listening (5%) were less prevalent. Note: Percentages referred to below under discussion of themes represent a percentage of the subset being discussed.

### 3.1. Themes

#### 3.1.1. Way of Being

Way of being, the listener’s conscious focus, intention, and manner as they engage with the speaker. Practitioner training sources emphasized that listening is an active, conscious process rather than a passive one (14.6% of the subset); listeners are to focus their attention in full (13.5%) to relate and connect with the speaker (8%).

During relational listening, understanding the listener’s perspective (11%), showing empathy (9%), and to a certain extent, curiosity (4%) become important elements in the way of being. Treating the speaker respectfully was a core sub-theme, entailing avoiding interrupting, talking over, or presenting counterarguments to the speaker (12%). Furthermore, listeners must suspend judgment and resist deeply analyzing their own reactions during the interaction in the moment (11%). The focus on the speaker, rather than the self, was important if the speaker’s perspective was to be understood (11%). Practitioners also recommended avoiding giving answers/solutions (2.3%), which, although less frequent, showed a significant level of agreement in the IRR analysis.

The final component of this theme referred to the listeners’ mindset, which guides attention to specific information. Some practitioners recommended listening more broadly for the overall message or story (5.5%) and fewer to listen for meaning on a human or interpersonal level (3%). Business or work-related texts analyzed (6.4%) recommended to focus on listening for accuracy or for facts, data, information, and accurate recall.

Only one or two sources recommended listening for “self-voice” (e.g., I, me) to gain insight into the speaker’s attributions. This sub-theme (known in the academic literature as active voice; Tannenbaum & Williams, 1968) was removed after IRR analysis due to low agreement. Following the discussion, it was deemed a specialist linguistic technique that mostly sits outside the framework of the lay practitioner’s listening training.

#### 3.1.2. Inner- Work

The listener can engage in preparatory work to prepare for upcoming conversations and develop into becoming a better listener. This theme reflects discussions of the inner- work or psychological strategies that a listener might apply during the conversation or prior to ensure that deep listening can occur. As a deliberate process, the listener considers what values drive their intention to listen (18%). The focus is on a benevolent intention; for example, the listener can embrace humility or opt to learn from and connect with the speaker.

The listener also maintains a mindful presence in the moment (17%), where they intentionally work to relax their personal defenses. This requires raising prior awareness of one’s own biases, beliefs, or feelings (20%) and one’s personal objectives, interests, or agenda (15.4%) so that these do not end up creating a distraction during the interaction. Bad habits that might obstruct listening (e.g., impatience, distractibility) should be planned to overcome (13.6%) and are recommended to be developed outside of the listening interaction and not during the conversation.

A few advanced training materials discussed the importance of developing personal courage (8%) and feeling safe to be vulnerable as a way of facilitating the possibility of changing one’s views (2.5%), which is a potential outcome of listening well. It was recommended that listeners engage in such preliminary internal work to prepare themselves for the challenges of hearing messages they might not want to hear (5%).

#### 3.1.3. Listening Behaviors

Observable listening behaviors that signal high-quality listening. The most common factors within this theme included the listener’s body language and facial expressions (16%), reflecting what the listener has heard (e.g., paraphrasing and summarizing) (15.4%), asking questions (15%), offering verbal cues to indicate listening (12%), and reflecting the speaker’s body language (7%). Many training activities were focused on practicing these skills. Asking follow-up questions was understood as helping to convey understanding or demonstrate that the listener has accurately attended to, recalled, and interpreted what the speaker has communicated. Verbal cues include affirmations such as “uh-huh” and “yes,” but also the use of silence and pauses (4%) to match the speaker’s pace. To provide full attention, listeners should remove distractions such as mobile phones (10%). A moderate amount of practitioner resources (9%) emphasized giving constructive feedback (e.g., “that’s great news”) and acknowledging or validating (5%) the listener’s message (e.g., “I see,” “makes sense”). Showing behaviors that express care, build trust, and create rapport (e.g., asking how the speaker is, reassuring that you care) was also recommended (6%).

A very limited number of resources focused on encouraging “storytelling” as a specific narrative technique. Only one resource recommended disclosing similar experiences to show understanding as a technique to connect with the speaker, while several others advised the contrary, to reserve self-disclosure. None of the trainings picked up on points highlighted in the primary articles used for a priori coding, addressing power imbalances between the speaker and listener to remove impediments to trust and openness (Kasriel, 2022) and reflecting muted or amplified emotions (Passmore, 2011). These four sub-themes presented with low IRR. As a result, they were excluded from the final interpretation of findings. It was agreed between the coding team that some of these could be considered advanced techniques.

#### 3.1.4. Holistic Listening

Holistic listening, attuning to less overt communication signals, and identifying incongruence with overt signals to intuit the real message. This theme highlights elements of listening training that encompass a holistic interpretation of the speaker’s communication, and in particular, moving beyond the surface-level interpretation of body language and verbal expression to identify underlying or unsurfaced emotions and messages.

The codes within this theme were less frequent than in other themes (representing only 5% of the dataset), suggesting that they are either reserved for more advanced audiences or emerging as a trend in lay practitioner training. We decided to include the theme holistic listening despite the lower prevalence based on guidance by Braun and Clarke (2021) that frequency of codes should not be a sole driver of themes, rather researchers should reflect upon relevance to the research question and quality of the theme. The codes in this theme presented a high IRR signifying consistency in coding, and relevance was further supported in discussions between coders, giving us confidence to report it as a theme.

The most commonly occurring sub-theme involved omissions; noticing what is not being spoken about explicitly (34% of the subset). For example, a person may share how much they enjoy the traveling or relocation requirements of their job, but they may omit a less obvious downside, such as missing family. In this example, a good listener might have a hunch (implicitly identified) by combining this “common sense” knowledge with noticing signs of sadness in the speaker.

Another sub-theme was incongruence or recognizing the underlying emotions of the speaker despite seemingly contradictory body language or verbal expression (27% of the subset of data). For example, a speaker might verbally express that they are excited to act, but their non-verbal behavior reflects low energy, apathy, or disengagement. In this case, the listener learns more about the speaker from attending to their body language than their words.

Noticing verbal nuances was another common sub-theme under this theme (21% of the subset of data) such as hyperbole (exaggerated expression or terms not meant to be taken literally, such as “I’ve said it a 100 times”), metaphors (e.g., “climbed a mountain”), or figurative speech (e.g., “opportunity knocking”) to consider again whether an underlying emotion or message is sitting behind this use of language. Use of such terms may also indicate how important or intense an experience might have been for someone. A final sub-theme focused on understanding the true meaning of words, considering a deeper meaning of what is being communicated beyond surface-level interpretation (19% of the subset). People may substitute words for something less direct out of politeness or because they do not feel they can communicate authentically (e.g., using the word “interesting” when they really mean “strange” as a negative reaction). The listener must interpret the speaker’s verbal intonations and nuances (e.g., sarcasm, politeness) and often, combine this information with the listener’s own knowledge and experience (e.g., idioms often have cultural associations). Together, these sub-themes form holistic listening.

#### 3.1.5. Training Techniques

Training techniques, content, and features of listening training design. The final theme addresses elements of the training design that support the development and training of listening. The most common elements included sharing examples of poor listening, contrasting against good listening (15%), the opportunity to practice using role-play or exercises (15%), addressing common barriers to listening, such as filtering, advising, etc. (13%), and sharing tips for common verbal cues and responses to indicate listening (8.5%). Incorporating the experience of feeling deeply listened to (5%) and discussion-based learning (2%) were also presented as training techniques. Two themes, reflective questions (4.6%) and pacing training to allow time for reflection (5%), both presented with low IRR, and following discussion, the analyst team agreed to merge these themes with discussion-based learning, as it was agreed there was overlap between these three sub-themes.

Formally measuring or assessing listening effectiveness was recommended by 5% of sources, for example, by using “The Listening Profile” adapted from Brownell (1996). Some sources described the psychology of listening (2%), but a larger number described the auditory, physiological process of hearing (11.4%). However, it could be argued that the psychology of listening overlaps with way of being and inner-work themes, even if they aren’t explicitly referred to as “the psychology of listening”. A small number of training materials included ideas for staying focused (4%), formulating a plan for listening in advance (2.4%), considering when it is appropriate to engage in active listening versus not (2.4%), how to encourage others to listen well (2.2%), and finally, noting cultural differences (2.4%).

### 3.2. Critical Analysis of Themes

**Figure 3:**
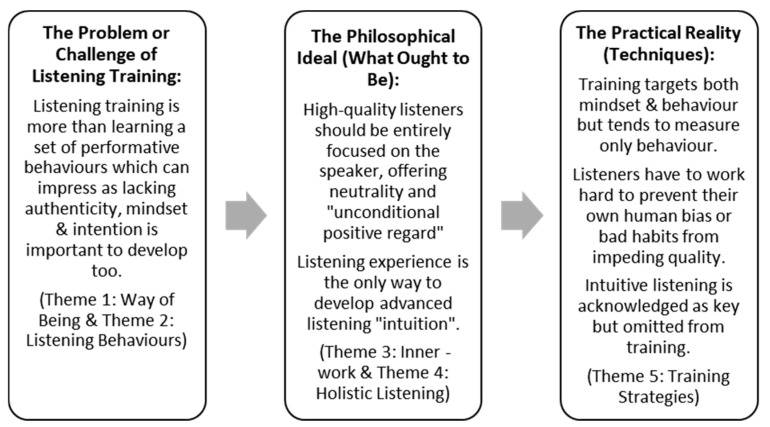
Critical analysis of themes.

In our analysis, inspired by the principles of GPT, which include identifying problems, techniques, and situated ideals (Craig & Tracy, 2014), we started with the problem or challenge of listening training. We juxtaposed philosophical ideals sought by listening practitioners (i.e., what ought to be) when learning/performing high-quality listening against what is and the logical, practical truth of what occurs during listening training (see Figure 3).

## 4. Discussion

Integrating recommendations from listening training sources provided by practitioners on the web, we sought to utilize this largely untapped data source further to inform listening theory, research, practice, and training. Five themes were identified that reflected both the internal process of listeners and their relational behaviors. These were termed way of being, inner-work, listening behaviors, holistic listening, and training techniques. A critical analysis (inspired by GPT; Craig & Tracy, 2014) of the themes highlighted a number of tensions between the practical application of listening training and key philosophical ideals that drive high-quality listening. These tensions are discussed in further detail below and presented as “dialectical listening theory” (DLT), alongside strategies practitioners employ to overcome or address these challenges and relevant psychological theory.

### 4.1. Tensions Experienced in Cultivating Listening

**Figure 4:**
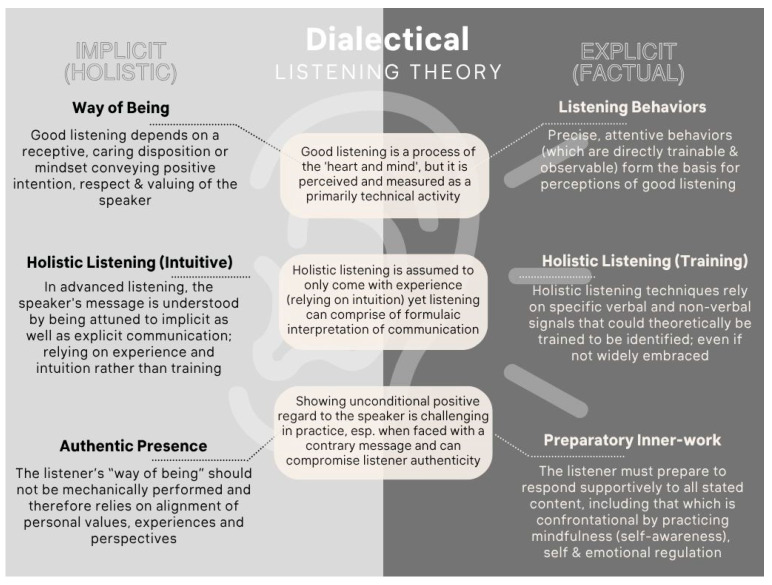
Dialectical listening theory (DLT)—three tensions in learning to listen.

Themes revealed three interesting tensions (see Figure 4) in the practice of listening and listening philosophy: (i) a listener can learn to perform the technical behaviors and verbal cues, but listening in this way may not compare well with listening that is driven by a specific mindset or “way of being”; (ii) listening very well (holistically) is more than a set of behaviors and relies on a “sixth sense” or intuition in addition to a way of being. Listening, therefore, comes with time and experience (versus training alone) despite the presence of formulaic techniques; and (iii) maintaining a listening “way of being” and “unconditional positive regard” can be challenging in the moment and has the potential to compromise listener authenticity, especially when faced with confronting or contrary messages.

We summarize these tensions as a dialectical listening theory (DLT). At a basic level, an excessive focus on technical performance detracts from an ideal mindset, compromising the quality of listening. Listening performance may be further challenged for listeners who fail to intuit the underlying meanings accurately from explicit expressions, and finally, good listening may be difficult to maintain when faced with messages that directly challenge or conflict with our own beliefs and values. On the whole, findings support that efforts in technical mastery can improve the listener’s effectiveness, but this alone is unlikely to suffice in generating positive downstream effects from high-quality relational listening.

These tensions present us with an overarching dialectic or paradox during high-quality listening; at an advanced level, implicit (or intuitive) processing of speaker communication is a key driver of listening, supporting greater authenticity on the one hand. Yet, when we stray away from explicit (or deliberate) processing of information (i.e., deeper processing of verbal cues by the listener; Burleson, 2011), our natural human tendency to convey personal biases or assumptions can impede non-judgmental listening, exacerbated when faced with conversations that conflict with one’s own perspectives or values. Good listening, then, may compromise listener authenticity as we actively seek to suppress our own judgments. It appears that good listening demands both implicit (intuitive) and explicit (deliberate) processes (Bodie & Jones, 2021), which may sit in conflict with each other at various stages of the listening process.

One dual-process model that best describes this phenomenon is Interactive Cognitive Subsystems (ICS; Barnard & Teasdale, 1991), a multi-level theory of human cognition and emotion which is considered a meta-theory of cognition because it aims to explain and incorporate many existing cognitive systems and theories (Teasdale & Barnard, 1993). ICS describes a cognitive structure comprising sensory inputs (visual, auditory, somatic), which lead to internal subsystems—a “mind’s eye” and “mind’s ear” where inputs are conceptualized and stored in memory. These are then processed in two central “meaning-making” subsystems of cognition; the first—described as a propositional meaning-making subsystem—takes information from the external world and processes input in an “intellectual” (explicit, factual) manner—for example, a good listener makes eye contact, asks questions, and nods on occasion. This system links in a back and forth manner to a second layer of cognitive processing referred to as the implicational meaning-making system where information is processed in a more “emotional” (implicit, holistic) manner—for example, it is here that the listener’s individual reactions are processed more holistically as a schematic model; the speaker may notice that despite the listener demonstrating the right behaviors, something feels “off”. They may be mechanistically performing the behaviors, but the emotional feeling is not resonating, compromising the listener’s way of being. Indeed, the overall implicational meaning may be different than the individual components (e.g., a somatic sensation combined with a propositional meaning) from which they are contrived (Teasdale & Barnard, 1993).

Another way of thinking about these two levels of cognitive processing is that the first (propositional) is “knowing with the head” and the second (implicational) is described as “knowing with the heart” (Barnard & Teasdale, 1991, p. 21). The propositional sub-system is linked to verbal and muscular/skeletal output (i.e., what we say and do), and the second layer results in somatic and visceral emotional reactions derived from the implicit, holistic processing of information. This is where intuition is derived from and explains the phenomenon of “gut-feel”, when someone has a sense or intuition about events, but they are unable to explain it rationally. The presence of these two separate sub-systems of meaning-making—one propositional and the second implicational (the latter of which is argued not to be present in animals)—allows one to think or reflect while acting at the same time (Barnard et al., 2007).

When it comes to addressing the challenges or tensions summarized above, the analyses identified several strategies employed by listening trainers. This includes engaging in preparatory “inner-work” (or self-development) to listen well and learning to apply psychological strategies (such as mindfulness) “in the moment” to address the challenge of withholding judgment and maintaining an authentic focus during listening. The second tension of relying on intuited or implicit processes to demonstrate holistic listening is not proactively addressed in listening training, and from this dataset, it appears to rely solely on gaining maturity in listening “experience”. We discuss these potential resolutions further below in light of current and future academic research.

### 4.2. Managing the Tensions

#### 4.2.1. Technical Performance Versus Mindset

To manage the first tension, technical performance versus mindset, practitioners suggest developing a way of being—an overarching mindset and approach that provides the core foundation needed for the optimal listening experience aligning with the philosophy of “active listening”—popularized by the humanist approach of Carl Rogers in the 1950s: that listening is only effective when the person embraces their role as a source of love and support (Rogers & Farson, 1957). Rogers’ book titled “A Way of Being” (Rogers, 1980) was more broadly philosophical writing about human potential, but he also applied this term to high-quality empathic listening as part of the therapeutic process, suggesting that good listening comprises more than a set of behaviors. The tension is that good listening cannot simply be “parroted” or mechanistically performed through trained behaviors.

A wealth of research supports the view that active listening behavioral responses directly contribute towards the perception of listening by the speaker, for example, an automated, computer-driven social skills training (for populations diagnosed with autism spectrum disorder) based on analyses of head-nodding and back-channel responses alone predicted perceived listening skills at a correlation coefficient of over 0.43 (Tanaka et al., 2020). This can have downstream relational effects on speakers’ trust, intimacy, and closeness (Gearhart & Bodie, 2011; Kluger et al., 2021). Yet, there is conflicting evidence relating to whether behavior alone will suffice as good listening. Some researchers have found that viewing listening as simply mastery of skills or behavior can be detrimental to social relationships and reduce listening motivation (Garland, 1981; Lachica et al., 2021). As a parallel, when considering the use of technology or “robots” to mirror active listening and mimic a human quality (Johansson et al., 2016), we suggest that this may be limited in the interpersonal impact on the speakers being listened to. Furthermore, we are unclear whether ‘holistic listening’—discussed further below—can be (effectively) learned and expressed by humans, let alone artificial intelligence, particularly as research into combined processing of verbal and non-verbal cues of communication is still emerging (Zhang et al., 2021).

To address the question, should listening training programs focus on helping people to identify and develop their “way of being,” and if so, how? While some programs sometimes spontaneously target this explicitly (e.g., Kubota et al., 1997), it is worth noting that careful systematic approaches to developing listening behaviors can, by proxy, increase confidence and reduce anxiety of listeners (Hansen et al., 2002; Itzchakov, 2020; Nemec et al., 2017). Indeed, mastery in learning can, in turn, improve learner attitudes with more enduring results (Kulik et al., 1990). Briefer listening training has shown mixed results in improving listening attitude over and above listening ability (e.g., Behrs, 1994; Tatsumi et al., 2010), suggesting that developing a “way of being” through achieving mastery in listening behaviors might take time.

The theme training techniques further reflected practitioners’ emphasis on embracing both intention and behavior during training with strategies that suggest listening skills (behaviors) are better built alongside intention (way of being), supporting empirical views by (Itzchakov & Kluger, 2017; Kubota et al., 2004). While the non-scientific practitioner and layperson widely embrace the term “active listening” to describe good listening, some researchers (Kluger & Itzchakov, 2022; Tyler, 2011) argue that use of the term has morphed to focusing on only teachable behaviors (such as paraphrasing and reflecting) rather than the original essence of active listening, which relies on empathy and unconditional positive regard (Rogers, 1980). In an influential paper, Tyler (2011) analyzed 12 business training sources sourced from the www and found that materials lacked sufficient depth to capture the true intention behind Roger’s concept of active listening (Tyler, 2011). Interestingly, the frequency of codes in our analyses put the listening behaviors theme on par with the way of being theme. With the larger set of sources here, it was possible to observe that the spirit of active listening, as well as its techniques, remains alive in practitioner recommendations today.

In practice, we suggest explicitly developing the listener’s mindset by inviting reflection on what the listener is listening for and what purpose it serves, tying in with self-transcendent values. We also advocate the implementation of more intensive, paced training programs.

#### 4.2.2. Intuition Versus Training

A second issue identified with listening training centers on the concept of holistic listening. Originally proposed by Lipetz et al. (2020), while lay people can describe how good listening looks in terms of broken-down components, listening is broadly perceived holistically. The listener’s intention, way of being, and behavior all play a role in shaping the speaker’s holistic perception of feeling listened to; together, they may be “greater than the sum of their parts”. Through this theme, we extend the concept of holistic listening to emphasize the importance of a listener’s ability to perceive the speaker’s intended message as a whole (e.g., even if it is not explicitly communicated) through omitted information, inconsistent body language, etc., and possibly before it even enters the speaker’s conscious awareness. Despite the lower prevalence of holistic listening in this dataset, the strong IRR of these codes and agreement between coders highlighted its importance. Many of the resources analyzed were limited to training outlines for basic-level listening training, yet closer inspection of rejected codes in the analyses (that presented with low occurrence/IRR) points to the presence of a broader range of advanced listening techniques (e.g., recognizing linguistic patterns) to support holistic listening.

Holistic listening is not a new concept. For example, it is widely acknowledged in the therapeutic literature (e.g., Therapeutic Metacommunication; Kiesler, 1988; Gestalt Therapy; Perls et al., 1994). Beyond linguistic patterns, holistic communication supports that listeners interpret a combination of verbal and non-verbal cues together, yet communication research that extends beyond linguistic processing is only just emerging, revealing a gap in our understanding of how we interpret body language and verbal communication together (Beattie et al., 2014; Trujillo & Holler, 2023; Zhang et al., 2021).

Holistic listening is attributed an almost magical quality by practitioners, described as intuitive—relying on a “sixth sense”—that only comes with experience. The ephemeral nature of this theme might explain why only 5% of the dataset contributed to it, revealing that it is less prevalent in training materials targeted towards the layperson. Practitioners perceive it as a challenge to teach or train novice listeners how to listen at this level, and it seems that practitioners accept this level of listening is reserved for the more mature (experienced) listener (e.g., in coaching; van Nieuwerburgh, 2017; Passmore, 2011). Indeed, holistic listening is likely to be processed as an implicit cognitive process, rather than an explicit process, as explained earlier through the ICS dual-process model (Barnard & Teasdale, 1991). Such cognitive processing relies on previously formed heuristics, mental associations, and schemas—supporting the capacity to “integrate[s] different parts of the speaker’s talk into a working whole” (Bodie, 2011a, p. 279). Doing so may require the use of working memory (Janusik, 2005), synthesizing information (Aotani, 2011), and making inferences (Hauser, 1984) based on verbal intonations and nuances (Nemec et al., 2017).

On a practical level, we speculate that true holistic listening cannot be developed in a short time but must be practiced and, therefore, must be trained alongside the development of inner-work. Exercises or examples that demonstrate culturally relevant discrepancies between verbal and non-verbal behavior could be leveraged to advance understanding of holistic listening as a concept to be further reflected upon in ongoing supervision sessions, as the listener continues with their practice. As an advanced level of listening, we suggest that it deserves future attention by researchers in the context of relational listening.

The focus on implicit versus explicit processing at an advanced level of listening sits somewhat in contrast to previous suggestions by listening researchers, which conclude instead that advanced listening requires more considered (explicit) processing of information, while basic levels of listening rely more on automatic, surface-level (implicit) cognitive processes (Burleson, 2011). It may be that this contrast exists because of a distinction between everyday listening, such as in superficial conversations, which may happen with less conscious effort, and the effortful listening a trainee may exert when first attempting listening training. Questions of effort, deliberation, and intent will be fascinating to explore in future studies of listening that is deep and empathic, in contrast to listening within less personal conversations.

#### 4.2.3. Authenticity Versus Unconditional Positive Regard

A third challenge was identified: In practice, it is difficult to maintain a conscious, positive regard during listening, especially when speakers’ views differ from those of the listeners or conflict with their values (Adamu et al., 2022). Consequently, it may be challenging for the listener to remain authentic in their “way of being”. When listening deeply and seeing the world through another’s perspective, there is a risk to mental frameworks that make up “the self,” which may be challenged (Rogers & Farson, 1957). This may result in automatic, unhelpful, or obstructive responses by the listener, such as withdrawing from emotions, over-involvement, or improper recall to person-specific triggers, referred to as “countertransference” in the field of psychotherapy (Fauth, 2006), leading to an impeded relationship and therapeutic outcomes (Hayes et al., 2018). It was widely believed that countertransference is pathological and avoidable; however, more modern viewpoints accept it as a natural human response, one that can be managed or even leveraged to enhance understanding of the speaker (Gabbard, 2001). The question of whether such unconscious and biased responses can be managed or overcome, and how best to achieve this, is not only debated by countertransference researchers but also by researchers in unconscious or implicit bias training to support diversity, equity, and inclusion in organizations and communities (Noon, 2018; Schmader et al., 2022). One practical strategy proposed for overcoming countertransference is demonstrating good listening skills (Fauth, 2006). Yet, findings in our study suggest that being able to listen well is the result of having addressed the underlying conflicts in the first place.

This final tension is practically addressed by listening trainers in this study’s review through the theme of inner-work. On a technical level, inner-work sub-themes in the results represent components of mindfulness (Kabat-Zinn, 2005), integrated self-regulation (Weinstein et al., 2013), and integrated emotion regulation (Roth et al., 2019). Indeed, these are considered core components of managing countertransference (Gelso & Hayes, 2001) by psychotherapy researchers. Practitioners mobilize this through taking time to engage in preliminary internal work; preparing to listen by reflecting on one’s own emotions and perspective, acknowledging potential obstacles such as personal biases or bad habits, and practicing psychological strategies (e.g., clearing the mind, focusing, and emotion regulation). This preparatory work can help individuals to develop a strong internal foundation for non-judgmental responding prior to engaging in listening interactions. We suggest that these personal regulation strategies should be included in listening training programs, with sufficient time for self-development to occur (e.g., by allowing time for self-reflection and discussion-based, experiential learning).

Beyond this, mindfulness has also been shown to play a role in facilitating relational listening (Goh, 2012; Manusov et al., 2020; Wachs & Cordova, 2007) and to support listening training (Schaefer, 2018). For example, interpersonal mindfulness is a relational form of mindfulness consisting of presence, awareness of self and others, non-judgmental acceptance, and non-reactivity (Pratscher et al., 2019). The aforementioned meta-theory of cognition, ICS, explains mindfulness as occurring within the meaning-making subsystems of cognition. Mindfulness is considered a purposeful consideration of what a person is doing, or what a person is experiencing emotionally (i.e., what is happening in the propositional and implicational sub-systems) without judging or attempting to change it. Training to engage in meta-thinking or “mindful experiencing” of emotional reactions supports the reconstruction of mental schemas, particularly when they are disruptive or contributing towards psychologically disordered thinking, incorporating new information from the propositional subsystem (Teasdale & Chaskalson (Kulananda), 2011). It may be that the listener needs to oscillate between “dual-process” states of explicit, factual, and implicit, holistic thinking modes during listening—taking time to develop self-awareness and acknowledge automatic thoughts, emotions, and reactions before considering how to respond. Mindfulness may even support the listener to engage in both states of cognition concurrently (Teasdale & Chaskalson (Kulananda), 2011; Evans & Stanovich, 2013; Spunt, 2013). Thus, it can offer resolution to ‘in the moment’ challenges of good listening, helping to address the effects on listener bias (Burgess et al., 2017; Gibb et al., 2022; Kanter et al., 2020). In all, it is our suggestion that mindfulness training is important to include in listening training.

While there has been research conducted on dual-process thinking for listening to persuasive (Chaiken, 1980) and supportive messages (Burleson, 2009), we suggest that further exploring dual-processing in the context of listening to views that conflict with one’s values or attitudes, and the impact on perceptions of listener authenticity, bias, and self-awareness will provide important avenues for future study.

Finally, the findings in this study also point to an important consideration in assessing listening skills through observer ratings (e.g., Kluger & Bouskila-Yam, 2017; Trahan & Rockwell, 1999). Those observations are limited to listening behaviors, but we emphasize the importance of assessments that measure actual and perceived listening attitude, as well as perception of listening behavior (e.g., Bodie, 2011a; Mishima et al., 2000), particularly in the context of relational listening as a shared, social phenomenon between more than one party.

### 4.3. Future Research Informed by Dialectical Listening Theory

Together, these findings support that training people to listen well is more complex than learning a set of verbal and behavioral responses. While good listening can be demonstrated in this way (in some cases within a short space of time), listening also relies on establishing a positive and intentional mindset. At a more advanced level, processing of “holistic” communication signals from the listener needs to be developed, yet formulaic strategies to support this development could be identified and shared more readily rather than waiting for “the magic to happen” or intuition to set in. Indeed, it is often argued that intuition is not always accurate and so honing this in can benefit the trainee (e.g., Price et al., 2016). However, this would rely on research in the area of multimodal communication cues having advanced further than it currently stands (Zhang et al., 2021). Much research to date has focused on a single mode (linguistic) rather than multi-modal processing (which might include prosody, gestures, and mouth movement) of communication. Questions remain unanswered, for example, the extent to which people process information in natural conversation by relying on multi-modal cues and the dynamics of such cues (Zhang et al., 2021).

A key challenge that will benefit those learning to listen well is preparing how to maintain a facilitative and supportive mindset in the face of conflicting views or opinions shared by the speaker. Being human means having biases and opinions, and raising awareness of and managing our personal biases in the listening process is a skill that needs to be learned if people are to develop into being good listeners. This could be addressed through engaging in personal development (inner-work; e.g., learning what your biases are, how they relate to your values, and how you prefer to respond to those who differ from you constructively), and by learning psychological strategies such as mindfulness and emotion regulation to apply “in the moment”. While these strategies may already be employed by some practitioner listeners, such as psychotherapists, this practice could be further investigated for effectiveness by any professional who aims to demonstrate high-quality listening, for example, teachers, doctors, managers, and coaches.

Learning to overcome this final tension in listening has a practical utility beyond supporting practitioners to listen well. We advocate that this aspect of listening training might be ideal for supporting diversity, equity, and inclusion programs, which traditionally rely on methods such as “unconscious bias training” with questionable impact (Noon, 2018; The Behavioural Insights Team, 2020). Such listening training, which equips trainees with the skills needed to listen to opposing and diverse perspectives (e.g., Cumberland et al., 2021) without judgment and while maintaining respect, may be a suitable alternative approach to investigate in terms of achieving similar intended aims of diversity training. Indeed, listening can depolarize and foster less extreme attitudes (Itzchakov et al., 2020; Itzchakov & Kluger, 2017). Creating comprehensive listening training or incorporating listening into diversity and inclusion interventions could support acceptance between individuals from diverse backgrounds and enhance tolerance towards minority groups in society.

Results also hint that courage is needed to listen well, a quality that is recognized as important in high-conflict or polarized contexts, such as when listening to communities and oppressed social groups (e.g., Catlaw et al., 2014; Thill, 2009). The listener can face vulnerability in such situations, and there is some evidence to suggest that listening can reduce vulnerability (Dhaliwal & Harrower, 2009). Work already focuses on how an intentional, thoughtful listener creates an environment of psychological safety, where the speaker can express themselves without fear of repercussions (Edmondson, 2004; Sapra & Kumar, 2020). Further work could also explore how the listener can listen without fear of repercussions to themselves. To date, the role of psychological safety has been limited to effects on the speaker during listening (e.g., Castro et al., 2016, 2018; Fenniman, 2010; Itzchakov et al., 2023), not the listener’s experience and mindset.

The present study carries important implications for workplace communication. The relevance of such development is underscored by substantial global investment in the sector; the global soft skills training market was valued at 37.2 billion dollars in 2025, with communication and productivity training representing the largest segment, accounting for around 41.2% of the market (IMARC Group, 2025). In organizational settings, employees and managers are frequently required to listen under conditions of time pressure, hierarchical pressure, evaluation, and disagreement. Our findings suggest that effective workplace communication training should therefore attend not only to visible listening behaviors, but also to the internal regulation required to sustain attention, non-defensiveness, and respectful engagement during difficult conversations.

### 4.4. Constraints on Generality

The findings in this review should be interpreted within the context and considering the limitations of the data. Researchers were based in the United Kingdom, and all resources were written in English. Results are not generally representative of global practitioners or cultural differences, and we cannot make extrapolations for non-Western cultures. While there have been a few works testing listening in non-Western cultures (e.g., Imhof & Janusik, 2006; Purdy, 2000; Wood & Alford, 2022; Zohoori, 2013), there has been little focus overall on cross-cultural differences in listening (Kluger & Itzchakov, 2022). Future work could analyze cross-cultural training sources to address this gap in research.

## 5. Conclusions

This study employed a systematic qualitative review using thematic analysis to explore listening training as it is presented in practitioner training materials. The search resulted in 207 listening protocols, and the analysis resulted in five themes: way of being, inner-work, listening behaviors, holistic listening, and training techniques. Themes were critically examined to identify tensions or challenges in the listening training process.

Analyses addressed practical questions such as: Does listening training need to target the development of both attitude and behaviors? Can holistic listening be broken down into concrete strategies, allowing us to fast-track training of advanced relational (implicit) listening skills? And can inner-work (including developing courage, self-awareness, and emotion-regulation) alongside learning practical skills such as “mindfulness” lead to better listening, particularly during challenging conversations? Results suggest that such self-development activities are essential in addressing the core tension of maintaining listener focus, neutrality, and authenticity.

The dialectical tensions identified in this study are especially relevant for workplace communication, where employees, managers, and colleagues must listen effectively across feedback, disagreement, coordination, and support. Organizational listening training should therefore move beyond surface-level behavioral scripts to cultivate the mindset and inner-work required for authentic, psychologically safe engagement, particularly under conditions of hierarchy, time pressure, and conflicting perspectives.

These insights could enhance listening training programs and contribute to developing listening theory. We posit a new, dialectical listening theory which highlights three main tensions in learning to listen; the overarching theme being a pull between (dual-process) states of explicit, factual, and implicit, holistic thinking (Barnard & Teasdale, 1991). We suggest that the listener learns to navigate these two states in order to demonstrate effective listening, and future research could examine this further.

Expanding on the original research aim, beyond training people to listen to enhance professional performance, foster interpersonal connection, and support well-being, the research could also guide tools such as fit-for-purpose listening training, which aims to bridge divides across diverse groups of people to support diversity, equity, and inclusion. Yet, when developing listening skills, the listener must be aware of tensions in the learning process, which may directly stand in the way of high-quality listening if not resolved.

## Data Availability

The data and links to materials underlying this article are available in the Open Science Framework repository at: https://osf.io/ht2by/?view_only=b8dbaaf395d7484da7f494ae4c29501b, accessed on 6 October 2024.

## Supplementary Materials

The following supporting information can be downloaded at: https://www.mdpi.com/article/10.3390/bs16040572/s1, Table S1: Themes and Sub-themes; Table S2: Kappa Calculations Measuring Agreement between Code Ratings. References (Cohen, 1960; Gable et al., 2004; Passmore & Oades, 2014; Woodcock, 2010; Landis & Koch, 1977; Krippendorff, 2004; Hallgren, 2012; McHugh, 2012) are cited in the supplementary materials.

## Abbreviations

The following abbreviations are used in this manuscript:

www	World-wide Web
ST-TA	Structured Tabular Thematic Analysis (Robinson, 2022)
IRR	Inter-rater Reliability
CI	Confidence Interval
DLT	Dialectical Listening Theory
ICS	Interactive Cognitive Subsystems (Barnard & Teasdale, 1991)
