# Supplementary material for: Understanding and Cultivating Effective Listening: A Dialectical Theory of the Tensions Between Intuition and Behavior
Source: Behav Sci (Basel). 2026 Apr 10;16(4):572. doi: 10.3390/bs16040572 (PMC13114012; doi:10.3390/bs16040572)
Supplement: Supplementary file 1 [file behavsci-16-00572-s001.zip › behavsci-4133295-supplementary.pdf]

Understanding and Cultivating Effective Listening: A Dialectical Theory of the Tensions between Intuition and Behavior:

Supplementary Information including Analysis, Table of Themes (with definitions) and Inter-rater Reliability Kappa Calculations

Supplementary S1: Analysis & Coding Tables

We undertook steps of analysis following the Structured Tabular Thematic Analysis (ST-TA; Robinson, 2022) framework, which involves an eight-step process (outlined below) to develop, count, and test the occurrence of qualitative codes.

Phase A: Deductive – a priori code and theme selection. The first five papers analyzed were sources known to the researchers that comprehensively covered the topic of listening, including a business magazine article, professional magazine articles, a website article for practitioners, and an academic journal article targeted at practitioners. These papers were selected as they would cover a comprehensive range of listening factors for people in practice, and they helped us as researchers to get into the data:

1. (Business Magazine Article): Deep Listening - Emily Kasriel - Stanford Review (Kasriel, 2022).
2. (Professional Magazine): Positive psychology techniques: Active constructive responding – The Coaching Psychologist, 2014 (Gable et al., 2004, as cited in Passmore & Oades, 2014).
3. (Practitioner Web Resource): Practicing Empathic Listening – positivepsychology.com (n.d.) https://positivepsychology.com/empathic-listening/

4. (Professional Magazine): Motivational Interviewing techniques: Reflective listening – The Coaching Psychologist, 2011 (Passmore, 2011).
5. (Academic Journal) - The Listening Guide for Coaching: exploring qualitative, relational, voice-centered, evidence-based methodology for coaches, Coaching: An International Journal of Theory, Research, and Practice (Woodcock, 2010).

The following data synthesis approach was applied to these texts to form an a priori set of codes:

1. The content was read and re-read from the initial data set (five articles) and notes were recorded.
2. Codes (sub-themes) were created from the first data set, and a table was created in Excel. Analysts looked for frequently used terms, different terms that referred to the same underlying meaning and emphasis of terms (Owen, 1984) to generate these.
3. Themes were identified from the first data set, and codes were re-organized into themes.
4. Codes from one theme were compared with codes from other themes and critically reviewed to identify anomalies.
5. The first draft of a "code book" was documented and presented to a team of five coders, with an explanation for each code and its meaning.

Phase B: Deep immersion in the data. The following analysis was then carried out:

6. The remainder of the texts were read, re-read, and coded according to the initial code book by the first rater using a tabular method by adding a "1" in the column for that code if it appeared in the source.
7. New codes were added inductively as they were identified through subsequent sources.
8. The team of additional raters cross-coded at least ten of the initial set of sources each, overlapping with each other by at least five sources so that a portion of sources were double-coded.
9. Coders met to review and reach a consensus on overlapping sources as a form of calibration and to ensure a shared understanding of what codes represented.

Phase C: Inductive, developing revised themes in the context of and influenced by a priori themes.

10. A coding meeting was held between all coders, and coders held a reflexive discussion on codes, disagreements in ratings were discussed, and critical feedback was sought on the formation of codes and themes until a consensus was reached. No major changes were made to the themes during this stage. However, clarification was achieved on various codes as coders gained familiarity with the qualitative content, and some codes were moved to different themes following discussion and agreement.

Phase D: Tabulating codes/themes against data chunks.

11. The coding team each rated an additional 30 sources, overlapping with each other by five sources to ensure a portion of sources were double-coded yet again.

12. Data were entered into excel, entering "1" in the column against codes/sources.

Phase E: Agreement and Phase F: Exploring code/theme frequencies.

13. Researchers performed an inter-rater reliability analysis to estimate Kappa between the two sets of ratings for each code that was double-rated to measure the consistency of judgment.

14. Coders met again to critically discuss codes that fell below the threshold for moderate agreement (IRR > .40), and an agreement was reached to remove these codes based on disagreement and low frequency (or prevalence) in consideration of the research question.

15. Final themes, codes, and descriptions were revised based on coder discussions and consensus on retention, rejection, overlap, and meaning, and the title was agreed upon for each of the themes and codes.

Phase G: Thematic Map and Phase H: Producing the report.

16. The themes and sub-themes were mapped and re-ordered logically, as a collaborative task between coders, before being documented in a final report.

17. The final codebook detailing themes and sub-themes was documented and presented (see Supplementary – Table S1).

Inter-rater Reliability Analysis

An inter-rater reliability (IRR) analysis was performed to assess the degree to which coders consistently assigned ratings to codes from the qualitative data. Kappa coefficient (Cohen, 1960) was chosen to measure the level of agreement between the categorical (yes/no) ratings while correcting for an agreement that might occur due to chance (refer to Supplementary – Table S2).

We followed recommendations by Landis & Koch (1977) regarding the interpretation of Kappa**.** We did not deem it necessary to follow Krippendorff's higher bar for interpretation (Krippendorff, 2004) due to the low level of risk present in our research question and the subsequent interpretation of results (i.e., it does not place human lives at risk; Hallgren, 2012). Where kappa estimates fell below .61 to .80 (substantial agreement), coders met and critically discussed possible reasons for lower agreement. Following discussion, codes that showed at least moderate agreement (> .41) were either modified with tightened descriptions, merged with similar sub-themes, or retained as they were, based on the agreement between coders.

In cases with lower reliability (fair, slight, or chance agreement at >.40), we noticed that in some cases, there were only one or two occurrences of the code in the data. Hallgren (2012) explains the lower agreement as an issue of prevalence, where "distributions of observed ratings fall under one category of ratings at a much higher rate over another" (p.6), resulting in kappa estimates falling lower than is representative. In this instance, prevalence (or lack of) is meaningful in our results, suggesting deviation from "general" listening training and possibly straying into

more specialized listening applications. For example, our analyses showed that the following codes resulted in a "Fair Agreement" (.21-.40); *7. Listen for self-voice, 26. Encourage story-telling* and *39. Reflecting back muted or amplified emotions,* coders agreed that these could be considered "specialized" listening skills employed by highly trained professionals such as therapists. This would explain the lack of prevalence in our data (which largely included training intended for audiences much broader than professional therapists). We agreed to remove these codes for this reason.

We also discussed the remaining codes with "Fair Agreement"; *56. Reflection questions: What surprised you, what did you learn?* And *57. Spread training over time to allow space for reflection and practice* in the Training Techniques theme. Coders agreed with one another that these overlapped with other codes (*45. Discussion-based learning,* and *53. Practice and Role-playing*) and that *spread training over time* was present in design but mostly not made explicit. Therefore we agreed to remove code 56. And 57. And tighten up the description for overlapping codes.

Percentage agreement for codes *32. Address power imbalances, 38. Recognizing gas-lighting and lying* and *50. Match speaker and listener to topic,* needed to be calculated manually as the kappa estimate was unable to be computed due to the variable being a constant (explained by lack of prevalence and agreement). We calculated percentage agreement by dividing the number of agreement scores by the total number of scores (McHugh, 2012). Again, coders agreed that these codes could be removed for low agreement.

Finally, codes where Kappa estimates revealed “Agreement equivalent to chance” were discussed, and it was agreed that *25. Disclose similar experiences to show understanding* could also be removed. Coders discussed that whilst this occurred in one source, there were references to avoid doing this in others. Therefore there was conflict about whether this is recommended for high-quality listening.

The final code, *33. Co-create narrative*, was discussed as overlapping with *26. Encourage story-telling* (also with lower agreement), and it was agreed that this could be removed as it could be considered a specialized therapeutic skill.

To summarize, all codes where Kappa estimates fell between 0 and .41, revealing less than moderate agreement, were removed (a total of 10 codes).

Fair Agreement – 7, 26, 39, 56 and 57

Chance Agreement – 25 and 33

0% Agreement – 32, 38 and 50

Table S1: Themes and Sub-themes

(With quotations from sourced text to illustrate meaning)

The final iteration of themes and descriptions involved re-ordering the sub-themes logically, and tightening up the descriptions following the coder discussions in the earlier process.

The output is below:

Title	Description	Quotes
**THEME ONE: WAY OF BEING**	**The listener's conscious focus, intention, and manner as they engage with the speaker.**	
1. Actively listens	A conscious process that requires the listener to *choose* to listen to what is being communicated	"Listening, on the other hand, is purposeful and focused rather than accidental. As a result, it requires motivation and effort."<br><br>*Virtual Speech, Source 41*<br>"…'active listening'. This is where you make a conscious effort to hear not only the words that another person is saying but, more importantly, the complete message being communicated."<br>*Mindtools, Source 6*
3. Focuses on speaker	Looking at, and maintaining attention on the speaker	"An active listener focuses on their communication partner and is able to express interest and engage meaningfully in the conversation"<br>*Masterclass, Source 115*
9. Demonstrates respect	Listener shows respect (does not speak over or counter-argue, treats as oneself would expect to be treated etc.)	"I listened to what they had to say and considered it, they usually got on board because they knew they'd been respected and heard"<br>*New York times. Source 128*
23. Suspends judgment	Listener "suspends" critical thoughts that might enter their head (that don't signal immediate warnings), for the duration of the conversation	"it is 'impossible' to answer all these questions at the same time as you are listening… Instead, you have to be ready and willing to pay attention to the speaker's point of view and changes in direction, patiently waiting to see where she is leading you."<br><br>*The Three A's of Active Listening, Source 59*
2. Avoids giving answers/solutions	Listener is not thinking about, or offering answers or	"You cannot allow yourself to become distracted by whatever else may be going on around you, or by forming counter arguments

	solutions in response	while the other person is still speaking."<br>*Mindtools, Source 6*<br>"…there can be risks in suggesting solutions. It takes responsibility away from the other person. It implicitly disempowers the other person by saying: 'You can't solve the problem, but I am better/smarter/more worldly than you, so I have to do it for you'. This can make the person feel belittled or patro nised."<br>*Australian Family Physician, Source 50*
12. Understands perspective	Listener tries to grasp the point of view and perspective of the speaker	"If you go into the discussion with the main goal of understanding their perspective, free of any judgment, people will open up to you"<br>*New York Times, Source 128*
10. Conveys empathy	Listener attempts to understand the feelings of the speaker, and expresses empathy	"When we listen empathetically, we go beyond sympathy to seek a truer understand how others are feeling."<br>*Business communication, basic concepts and skills, Source 113*
14. Cultivates genuine curiosity	Listener channels curiosity into the direction of the speaker and is open to learning by listening	"Bad listeners make snap judgments that justify the decision to be inattentive. Yet, since you're already there, why not listen to see what you can learn?"<br><br>*The Three A's of Active Listening, Source 59*
4. Listens to relate	Listener engages with the intention to relate to the speaker, despite potential difficulties	"How to create understanding, trust and deeper connections with others through active listening skills".<br>*The Power of Deep Listening, Source 12*
8. Listens for social cues; in relation to people, groups or audiences	Listener attends to information that conveys the speaker's intention or understanding of social situations (including this interaction)	"focuses on what the interaction means for others. They filter what is heard through interests in other people, groups and audiences. They are socially intuitive and can pick up and respond to subtle cues."<br>*Mandel, Source 9*<br>"…using systematic reasoning and careful thought to analyze a speaker's message and separate fact from opinion. Critical listening is often useful in situations when speakers may have a certain agenda or goal, such as watching political debates, or when a salesperson is pitching a product or service."<br>*Maryville University, Source 26*
5. Listens for facts, data and information	Listener attends to information that is objective such as (facts, data or information)	"When we're listening to learn or be instructed we are taking in new information and facts, we are not criticising or analysing. Informational listening, especially in formal settings like in work meetings or

		while in education, is often accompanied by note taking…" *Skills You Need, Source 25*
6. Listens for overall message	Listener is able to pull away from the detail and comprehend the broader message being conveyed (e.g. message, plot. narrative. story, concept)	"Each time, the listener must try to remain sensitive to the total meaning the message has to the speaker. What is he trying to tell me? What does this mean to him? How does he see this situation?" *Gordon Training International, Source 60* "The key then is for the listener to quickly ascertain the speaker's central premise or controlling idea. Once this is done, it becomes easier for the listener to discern what is most important." *Three A's of Active Listening, Source 58*

**THEME TWO: INNER-WORK**	**The listener can engage in preparatory work to prepare for upcoming conversations and develop into becoming a better listener.**	
19. Undertakes preliminary internal work	Listener makes the time and commitment to do inner-work to prepare to listen well	"If you really want to be heard and understood by another, you can develop him as a potential listener, ready for new ideas, provided you can first develop yourself in these ways and sincerely listen with understanding and respect."<br><br>*Gordon Training International, Source 60*<br><br>"To completely empty oneself of ones own prejudices, patterns of responding and frame of reference, and to try to understand all of this about another person is an act of great generosity and respect. It is a commitment of not only time, but mental energy and a preparedness to explore another person's world and see the way life appears to them."<br><br>*Family Physician, Source 48*
21. Raises self-awareness	Considers own biases, feelings and beliefs on a topic	"In order to understand the need for active listening, we need to be aware that we receive and evaluate everything through our personal lens, through which we interpret the world."<br><br>*Positivepsychology.com, Source 58*<br><br>"We listen to what is going on within ourselves, as well as to what is taking place in the person we are hearing."<br><br>*Australian Family Physician, Source 50*

15. Addresses obstacles to good listening	Identifies and overcomes bad habits and listening preferences	"You will notice how hard can be to listen to a person and/or to a situation, especially when you don't agree, you feel bored or you are eager to express your opinion. You will also realize how practice is key!"<br>*Udemy Course, Source 14*<br><br>"Old habits are hard to break, and if your listening skills are as bad as many people's are, then you'll need to do a lot of work to break these bad habits."<br><br>*Mindtools, Source 6*
16. Identifies virtuous intention	Considers the virtues of listening well (e.g. humility, connection, understanding)	"The foundational component of the deep listening approach is how the listener shows up to the discussion—in terms of both their intention and the kind of attention they give to the speaker. Entering into conversations with humility is a simple yet potentially transformational way to help create more profound encounters."<br><br>*Deep Listening - Emily Kasriel, Source 1*
18. Sets aside personal agendas and interests	Considers own interests and questions in relation to satisfying own needs, and sets these aside for the purpose of the interaction	"You will always need to make up your own mind about where you stand —whether you agree or disagree with the speaker—but it is critical to do so after listening."<br><br>*The Three A's of Active Listening, Source 59*<br><br>"…approaching all conversations without any preconceptions of what might happen or what someone else might say. This allows you to listen without being critical and will stop you from getting distracted if any of your assumptions are proved wrong or right"<br><br>*Virtual College, Source 117*
17. Mindful practice	Practices mindfulness; learns how to be fully present and attentive to what the listener is saying	"Think of listening as a form of meditation. You have to clear your mind of everything else, so you can focus entirely on what the other person is saying"<br><br>*How to be a better listener – smarter living guides, Source 128*<br><br>"Sometimes the subject may be quite boring, force yourself to refocus. It is a skill and does require practice."<br><br>*Careers in Sport, Source 104*

		"In an interpersonal context, active listening aims to minimize the effect of our biases and to practice mindful patience whilst bypassing our own agenda".<br><br>*Positivepsychology.com, Source 58*
22. Considers vulnerability and authentic communication	Considers in advance how vulnerable and honest they can be to create feelings of safety and trust	"Getting in touch with our own feelings… Listening as gift and authentic Communication"<br><br>*Listen Well Scotland, Source 16*<br><br>"Listening expert Avraham Kluger also asserts that projecting honest vulnerability helps create a sense of safety: "I've learned that if I am brave enough to share a weakness, it demonstrates on some level that I have accepted myself, that I can accept others."<br><br>*Deep Listening - Emily Kasriel, Source 1*
13. Develops courage and accepts possibility to change	Works towards feeling secure and courageous enough to accept that their own views might be changed as a result of listening well	"Active listening carries a strong element of personal risk. If we manage to accomplish what we are describing here—to sense deeply the feeling of another person, to understand the meaning his experiences have for him, to see the world as he sees it—we risk being changed ourselves… To get the meaning which life has for him - we risk coming to see the world as he sees it. It is threatening to give up, even momentarily, what we believe and start thinking in someone else's terms. It takes a great deal of inner security and courage to be able to risk one's self in understanding another."<br><br>*Gordon Training Institute, Source 60*

**THEME THREE: LISTENING BEHAVIORS**	**Observable listening behaviors that signal high-quality listening.**	
20. Removes distractions in environment	Turns off cell phone, technology notifications, organizes a suitable private space etc.	"These distractions may be influencing, how much are advisors listening to what the customer actually wants?"<br><br>*Call Centre Helper, Source 130*
29. Listener's body-language	For example, mannerisms, breathing, posture, eye contact signal attention, smiles,	"Show that you are engaged and interested by making eye contact, nodding, facing the other person, and maintaining<br>An open and relaxed body posture. Avoid attending to distractions in your environment or checking

	avoids frowns, mirrors the speaker	your phone. Be mindful of your facial expressions: Avoid expressions that might communicate disapproval or disgust."<br><br>*Greater Good Science Center, Source 36*
30. Listener's verbal cues	For example, verbal affirmations such as "yes", "uh huh" to encourage the speaker	"… use innumerable verbal encouragers with minimal or no interruption and yet provides the patient with the necessary confidence to keep going. Such neutral facilitative comments include "uh-huh", "go on", "yes", "um", "I see" – we all have our own particular favourites."<br><br>*Listening Attentively – The Skills, Source 54*
11. Establishes rapport and trust	Listener makes an attempt to establish rapport, show care and build trust	"Build trust. As you speak to customers make sure you use an empathetic and friendly tone. While doing this let customers know that you're doing everything in your power to help them. They need to know that you are a champion here to defend them, not an obstacle in their way"<br>*Gladly customer service, Source 125*
31. Reflects back speech	Repeats back what the speaker has said through summarizing and paraphrasing salient points that have been understood. This requires remembering what the participant has said.	"Reflecting is paraphrasing back to the speaker what they said. One of the things a lot of us find when we try to use this technique is that it's real a challenge. We don't want to just parrot back what was said; we want to paraphrase. It takes creativity to think of appropriate ways to paraphrase what we've heard"<br><br>*Scott Williams Listening Training, Source 123*<br><br>"Remembering details, ideas and concepts from previous conversations proves that attention was kept and is likely to encourage the speaker to continue".<br><br>*Virtual Speech, Source 41*
40. Reflects back emotions	Describes the speaker's emotions and energy to reflect what is being observed (e.g. your shoulders have really sunk as you said that)	"For example, if someone is sharing how they are sad about a lost pet, do not respond by talking about when this last happened to you. Instead, ask them a follow-up question to show that you care about their experience."<br><br>*Positivepsychology.com, Source 58*<br><br>"the listener has to summarise the key things described by the talker including facts about what was described as well as emotions that were described."

		Communication Exercises, Source 65
27. Asks follow-up questions	Asks open-ended questions aim to learn more about what the speaker is saying (e.g. Can you tell me more about…?) while avoiding *why* questions. Clarifying questions aim to ensure correct understanding of what has been heard (e.g. did I get that correct?)	"Ask clarifying questions to gain more information. You can also ask confirming questions, such as "I want to make sure I got that right. It sounds like you're saying Is that correct?" This can help you gauge if you've received the message accurately. "<br><br>*Maryville University, Source 26*
24. Offers acknowledgement or validation	Recognizes the feelings and emotions of the speaker in an affirming way by offering verbal acknowledgement or validation of what has been spoken.	"…give verbal affirmations to show that you understand what the speaker is telling you. Saying things like 'yes' and 'I see' or 'you're right' lets the person talking know that you are following what they're saying and makes them feel more confident and at ease."<br><br>*Virtual College, Source 117*<br><br>"Show your attentiveness using sentences such as "I can imagine how sad you must have been," or in a happy update, "I hope you are impressed with yourself!"<br><br>*Positivepyschology.com, Source 58*
28. Gives constructive feedback	Moving beyond acknowledgment and validation, offers personal feedback that is supportive in nature. For example, "that's great news that x has happened".	"Rather than simply acknowledging what has been said, the coach, using ACR [active constructing responding], responds to the excitement in the voice tone and body language by providing positive feedback ('I'm so pleased'). This response is grounded, supported by evidence ('because you have worked so hard over the past months, and you deserve this…well done….I'm so proud of you')."<br><br>*Positive Psychology Techniques – Active Constructive Responding, Passmore & Oades (2014), Source 2.*
36. Matches thinking pace of speaker	Shows patience, adjusts pace of conversation to suit the speaker and allows for noticeable periods	"Resist the urge to fill moments of silence. There are different types of silence. Respecting quiet moments can a powerful tool for a deep conversation. It gives the speaker and receiver a chance to reflect and continue with this process. So often we rush to "fill"

	of silence to allow the speaker time to think and reflect.	silence, right before someone has a breakthrough thought to share."<br><br>*Three A's of Active Listening, Source 58*<br><br>"Silence is also a powerful tool for both speaker and listener. In some East Asian cultures, for example, silence is a sign of respect for what has been said and it would seem rude to immediately speak after the other person has finished. It's also important to remember colleagues who speak your language as their second or even third language sometimes need longer to formulate their thoughts. Pausing is thinking time rather than a signal for you to start talking."<br><br>*Active Listening across Cultures, Source 23*

**THEME FOUR: INTUITIVE LISTENING**	**Attunes to less overt communication signals and identifies incongruence with overt signals to intuit the real message.**	
34. Considers omissions (what isn't being spoken about explicitly)	Considers what is not being spoken about directly, or what is not being said, to correctly interpret the entire message or meaning of what is being spoken.	"A key to understanding communication is not only listening to what is said, but also to what is *not* said. The ability to read between the lines and tune in to non-verbal signals, such as facial expressions and body language, can help significantly"<br><br>*Active Listening Across Cultures, Source 23*
35. Notices incongruence between speaker's overt communication and body language	Understands the speaker's true emotions, despite seemingly contractionary body language, verbal message etc.	"In some instances, the content is far less important than the feeling which underlies it. To catch the full flavor or meaning of the message, one must respond particularly to the feeling component."<br>*Gordon Training International, Source 60*<br>"When the feelings, body language, or voice expression do not match the verbal message, this is called incongruence. The speaker is sending conflicting signals… Some examples of responses from a listener might be, 'You say you want to get home early, but your voice sounds hesitant."<br><br>*Student Manual - Listening for Understanding, Source 38*
41. Considers true meaning of words	Considers whether specific words used accurately reflect, and are consistent with the broader,	"… often use vague or tentative language when they speak, using word choices that may not accurately reflect what they mean. Depending on the context, there could be several reasons for this. It might mean that they are unsure of what they want to say, and

	intended message of the speaker	are having trouble expressing themselves; or it might mean that they are uncomfortable with the topic."<br><br>*Paraphrasing in a Nutshell, Source 56.*
37. Notices and considers the speaker's verbal nuances	Notices, and looks for patterns in the speaker's use of words or images. For example, using metaphors, hyperbole, superlatives, figurative language.	"Repeated words, phrases and images, information and comments that jump out at the coach, contradictions, omissions, and revisions… helping… follow… what is meaningful to the client"<br><br>*Woodcock, Source 5*<br><br>Listening implies decoding (i.e., translating the symbols into meaning) and interpreting the messages correctly in communication process.<br><br>*Student Listening, Source 36*

**THEME FIVE: TRAINING TECHNIQUES**	**Content and features of listening training design.**	
52. Explains the physiological aspects of listening (hearing vs listening)	Explains that hearing is physiological, recognizing that something is being said - different to listening, which is "internalizing" or processing the words of the speaker.	"Hearing and listening are not the same. You hear music, the sound of rainfall, or the sound of food being prepared in the kitchen. Listening, on the other hand, requires attention, comprehension of the message that's being relayed, and recollection of what's been said."<br><br>*Maryville University, Source 27*
54. Explains the psychology of listening	Explains psychological theories and processes related to listening	"Some of the powerful topics you'll discover include: ✓The psychology of listening"<br><br>*Listening skills training, Source 15*
55. Discusses when to and when not to engage in active listening	Recognizes when it is appropriate to listen and when it's not (e.g. medical context)	"There may be legitimate reasons why it is inappropriate to actively listen in any given situation, but rather than deny the need, it is usually more helpful to acknowledge it, and arrange a more appropriate time or setting to address it."<br><br>*Australian Family Physician, Source 48*
42. Explores barriers to effective listening	Explains common obstacles to listening effectively, for example,	"Some of the factors that interfere with good listening might exist beyond our control, but others are manageable. It's helpful to be aware of these factors so that they interfere as little as possible with

	rehearsing, filtering, advising, attention span	understanding the message. Here are some key barriers: 1. Noise, 2. Attention Span…”<br><br>*Virtual Speech, Source 41*<br><br>“There are a multitude of factors that may impede upon someone's ability to listen with purpose and intention; these factors are referred to as listening blocks.[14] Some examples of these blocks include rehearsing, filtering, and advising.”<br><br>*Wikipedia, Source 61*
44. Explores or considers cultural differences in listening	Raises awareness of cultural differences and discusses examples or encourages exploration of how to navigate these	“Be aware of how certain differences (such as gender, race, age, authority, language) between you and the other person might impact how each is perceived by the other, or how each person might perceive the other.”<br><br>*Workplace Learning and Development – Umass Amherst, Source 22*<br><br>“Always look directly at the speaker. In some countries, direct eye-contact is rude and offensive. However, in Western Culture, direct eye contact means you are listening.”<br><br>*Listening for Understanding, Source 38*
46. Shares examples of good and poor listening	Runs through practical examples of what good and bad listening behaviors look like to demonstrate and contrast	For example:<br><br>“Nod your head, smile and make small noises like “yes” and “uh huh”, to show that you’re listening and encourage the speaker to continue. Don’t look at your watch, fidget or play with your hair or fingernails.”<br><br>*British Heart Foundation, Source 119*
58. Shares tips for responding and good listening	Includes examples of phrases and questions that a good listener might say to demonstrate that they are listening well	For example:<br><br>“In active listening, open-ended questions are questions that cannot be answered with ‘YES’ or ‘NO’. Examples of open-ended questions include:<br>What alternatives have you thought about... ?<br>What do you mean by... ? …<br>Examples of paraphrasing statements include:<br>I’m not sure I’m with you but...<br>If I’m hearing you correctly...”<br><br>*The University of Adelaide, Source 42*
49. Shares ideas for staying focused	Offers psychological	“If you're finding it difficult to focus on what someone is saying, try repeating their words in your

	strategies for how to stay focused and avoid distractions during listening	head as they say them – this will reinforce what they're saying and help you to concentrate. Try to shut out distractions like other conversations going on in the room. And definitely don't look at your phone."<br><br>*British Heart Foundation, Source 119*<br><br>"Tip:<br>If you're finding it particularly difficult to concentrate on what someone is saying, try repeating their words mentally as they say them. This will reinforce their message and help you to stay focused."<br><br>*Mindtools, Source 6*
48. Explores how to encourage listening in others	Addresses situations where one might be engaging with someone who is not a good listener	"What if you are the one speaking and the other person isn't being an active listener? All of us have been in a situation where the person listening to us was distracted or disinterested. The following are some tips to help you with this situation… In doing so, you might help that person learn how to become a better listener."<br><br>*Very Well Mind, Source 63*
43. Develops a plan for good listening	Encourages preparation of a plan detailing techniques to be used in a situation where good listening is required	"Lead a discussion in which students develop a code of listening behavior (see Activity 4) for their classroom".<br><br>*Listening Activities, Source 45*
53. Allows time for practice and role-playing	Allows time and space for practicing listening, or role-playing in listening scenarios (good and bad)	"skills training also gives the learners rehearsal space"<br><br>*Flick Learning, Source 70*<br><br>"Ask two pairs of students to demonstrate for the class both poor and good listening skills. Tell students to observe you in the conversation."<br><br>*United States Institute of Peace, Source 52*
47. Incorporates experiential learning activities	Gives the listener the experience of being deeply listened to by someone who can listen well so that	"Participants had brief conversations (about their biggest disappointment with their university) with someone trained to engage in active listening, someone who gave them advice, or someone who gave simple acknowledgments of their point of view. Participants who received active listening reported

	they understand what it feels like	feeling more understood at the end of the conversation".<br><br>*Greater Good, Source 36*
45. Discussion based learning	Allows time and space for reflection and discussion to facilitate learning	"3. At the end of the conversation, ask the student how he or she felt while they were talking.<br>4. Ask the class what listening skills, good or bad, that they observed.<br>5. Explain to the class that good listening requires active participation. Ask students for examples of how to be a good listener. Write these on the board, separating the verbal and non-verbal skills…<br>8. Lead a class discussion using some or all of the following questions:<br>• How did you know that your partner was listening to you?<br>• What did it feel like to really be listened to without being interrupted?<br>• What made this activity challenging for you?<br>• How can active listening help you resolve conflicts?"<br><br>*Institute of Peace, Source 51*
51. Measures listening effectiveness (e.g. through assessment)	Incorporates a form of listening evaluation or measurement to determine whether listening has improved	"Why you'll love our courses…<br>- Multiple choice exam with an 80% pass mark<br>- Unlimited exam retakes at no extra cost"<br><br>*Active Listening online training course, Source 76*<br><br>"The following are possible means to evaluate student mastery of the objective and standards addressed in this lesson.<br>1. Differentiate between hearing and active listening.<br>2. Replay Active Listening Kahoot! with improvement in answering the six questions.<br>3. Complete SKILFUL listening questionnaire compare to the one completed before the lesson. Ask students if they rated themselves differently after completing this lesson."<br><br>*Illinois State University, Source 38*

Table S2: Kappa Calculations Measuring Agreement between Code Ratings

The following tables reflect codes and themes *before* coder discussions and reviews that involved re-naming, re-ordering and moving of sub-themes to appropriate themes. The bolded sub-themes had substantial agreement or more in the IRR.

THEME ONE: WAY OF BEING	Kappa Value (*k* value)	Approx. Sig (*p*-value)	Interpretation	Frequency (first rater only)
1. Active Listening - conscious process	0.604	0.000*	Moderate Agreement	127
**3. Focus on the speaker**	**0.801**	**0.000***	**Substantial Agreement**	**117**
9. Respect (don't speak over, counter-argue, treat as you would expect to be treated etc.)	0.589	0.000*	Moderate Agreement	104
**12. Understand their perspective**	**0.634**	**0.000***	**Substantial Agreement**	**96**
10. Show empathy	0.607	0.000*	Moderate Agreement	76
4. Listen for connection with others	0.511	0.000*	Moderate Agreement	69
5. Listen for facts, data, and information	0.435	0.002*	Moderate Agreement	56
11. Trust, rapport, care (relationship)	0.537	0.000*	Moderate Agreement	51
6. Listen for the overall message, plot, narrative story, concept	0.564	0.000*	Moderate Agreement	48
8. Listen for social cues, people, groups, audiences, and what it means to others	0.557	0.000*	Moderate Agreement	25
**2. Avoid giving answers/solutions**	**0.628**	**0.000***	**Substantial Agreement**	**20**
7. Listen for self-voice (I, they, you etc.)	0.243	0.051	Fair Agreement	6

**significant at 95% confidence interval. Total Theme One Frequency: 791.*

THEME TWO: INNER- WORK	Kappa Value (*k* value)	Approx. Sig (*p*-value)	Interpretation	Frequency (first rater only)

23. Suspend judgment (vs. non-judgmental) through awareness	0.567	0.000*	Moderate Agreement	96
20. Remove distractions in the environment	0.48	0.000*	Moderate Agreement	86
**21. Self-awareness (biases, beliefs, feelings)**	**0.69**	**0.000***	**Substantial Agreement**	**57**
**16. Intention (e.g., humility, learn, connect)**	**0.648**	**0.000***	**Substantial Agreement**	**50**
17. Intentionality (mindful presence, let go of defenses)	0.5	0.000*	Moderate Agreement	48
**18. Park agendas**	**0.635**	**0.000***	**Substantial Agreement**	**43**
**15. Identify and overcome bad habits and listening preferences**	**0.677**	**0.000***	**Substantial Agreement**	**38**
**14. Genuine curiosity**	**0.67**	**0.000***	**Substantial Agreement**	**35**
**13. Feeling secure and courageous to sit with discomfort and the possibility to change.**	**0.847**	**0.000***	**Near Perfect Agreement**	**22**
19. Preliminary internal work	0.541	0.000*	Moderate Agreement	14
22. Showing vulnerability creates psychological safety (trust self)	0.48	0.001*	Moderate Agreement	7

**significant at 95% confidence interval. Total Theme Two Frequency: 486.*

**THEME THREE: BASIC LISTENING ACTIONS**	**Kappa Value (*k* value)**	**Approx. Sig (*p*-value)**	**Interpretation**	**Frequency (first rater only)**
**29. Listener body language (frowns, smiles, mannerisms, breathing, posture, eye contact, mirror)**	**0.75**	**0.000***	**Substantial Agreement**	**136**
31. Reflecting through summarising/paraphrasing - salient points, remembering what has been said	0.424	0.002*	Moderate Agreement	131
27. Follow up questions – e.g. did I get that correct? Tell me more? Have I missed anything? Avoid why. Open-ended.	0.494	0.000*	Moderate Agreement	129
**30. Listener's verbal cues, including silence, pause**	**0.645**	**0.000***	**Substantial Agreement**	**105**
28. Giving Feedback (e.g., that's great news that x has happened)	0.531	0.000*	Moderate Agreement	78

24. Acknowledgement/validation	0.564	0.000*	Moderate Agreement	42
26. Encourage storytelling	0.397	0.002*	Fair Agreement	11
25. Disclose similar experiences to show understanding	-0.027	0.838	Agreement equivalent to chance	11

**significant at 95% confidence interval. Total Theme Three Frequency: 637.*

**THEME FOUR: ADVANCED LISTENING TECHNIQUES**	**Kappa Value (*k* value)**	**Approx. Sig (*p*-value)**	**Interpretation**	**Frequency (first rater only)**
40. Reflect back on interpretation and energy of speakers emotions (e.g., by describing body language)	0.607	0.000*	Moderate Agreement	60
34. Interpretative listening: Consider the deeper narrative (what isn't being spoken explicitly)	0.547	0.000*	Moderate Agreement	44
39. Reflect back (muted) (e.g., tone down the emotion or reflect back (amplified) emotion)	0.293	0.028	Fair Agreement	36
35. Intuition of speaker's emotions	0.453	0.001*	Moderate Agreement	35
**36. match the thinking pace of the speaker (i.e., patience)**	**0.673**	**0.000***	**Substantial Agreement**	**33**
**37. Notice /emphasize the speaker's verbal nuances, figurative, metaphor, hyperbole, superlatives**	**0.733**	**0.000***	**Substantial Agreement**	**27**
**41. True meaning of words**	**0.807**	**0.000***	**Substantial Agreement**	**25**
33. Co-create narrative	-0.02	0.886	Agreement equivalent to chance	6
38. Recognizing gas-lighting and lying	Not Calculated- variable is constant	Not Calculated – variable is constant	% Agreement calculated manually = 0	3
32. Address power imbalances - motivations that impede trust and openness – For example, show that lived experience is more valuable than power, Ethics - give speaker control	Not Calculated- variable is constant	Not Calculated- variable is constant	% Agreement calculated manually = 0	2

**significant at 95% confidence interval. Total Theme Four Frequency: 264*

**THEME FIVE: TRAINING TECHNIQUES**	**Kappa Value (*k* value)**	**Approx. Sig (*p*-value)**	**Interpretation**	**Frequency (first rater only)**
46. Examples of good/poor listening to contrast	0.448	0.001*	Moderate Agreement	60
53. Practice and Role-playing	0.522	0.000*	Moderate Agreement	60
**42. Barriers to listening (e.g., rehearsing, filtering, advising)**	**0.657**	**0.000***	**Substantial Agreement**	**54**
52. Physiological aspects of listening (hearing vs. listening)	0.495	0.000*	Moderate Agreement	47
58. Tips for responding	0.508	0.000*	Moderate Agreement	35
**51. Measure listening effectiveness (e.g., assessment)**	**0.622**	**0.000***	**Substantial Agreement**	**22**
47. Give the listener the experience of being deeply listened to so that they can embody and understand it.	0.491	0.000*	Moderate Agreement	22
57. Spread training over time to allow space for reflection and practice	0.156	0.232	Fair Agreement	20
56. Reflection questions: What surprised you? What did you learn?	0.194	0.019*	Fair Agreement	19
**49. Ideas for staying focused**	**0.79**	**0.000***	**Substantial Agreement**	**16**
**55. Recognize when it is appropriate to listen and when it's not (e.g., medical context)**	**0.658**	**0.000***	**Substantial Agreement**	**10**
**44. Cultural differences**	**0.73**	**0.000***	**Substantial Agreement**	**10**
**43. Create a plan**	**0.79**	**0.000***	**Substantial Agreement**	**10**
48. How to encourage listening to others	0.469	0.000*	Moderate Agreement	9
54. Psychology of Listening	0.48	0.001*	Moderate Agreement	9
45. Discussion-based learning	0.485	0.000*	Moderate Agreement	9
50. Match speaker and listener to the topic (e.g., racial equity topic, then same race)	Not Calculated- variable is constant	Not Calculated- variable is constant	% Agreement calculated manually = 0	7

**significant at 95% confidence interval. Total Theme Five Frequency: 415.*
